# Unveiling the Synergy of Serum Lipoprotein-Associated Phospholipase A2 and PLA2G7 Gene Polymorphism (rs1805017) as Key Determinants of Coronary Artery Disease Risk and Severity: Implications for Early Intervention

**DOI:** 10.7759/cureus.74045

**Published:** 2024-11-19

**Authors:** Hajra Luqman, Noorjahan Mohammed, Iyyapu Krishna Mohan, Kompella S. S Saibaba, Oruganti Sai Satish, Madrol Vijaya Bhaskar, Neelam N Sreedevi, Siraj A Khan

**Affiliations:** 1 Biochemistry, Nizam's Institute of Medical Sciences, Hyderabad, IND; 2 Cardiology, Nizam's Institute of Medical Sciences, Hyderabad, IND; 3 Biochemistry, Nizam’s Institute of Medical Sciences, Hyderabad, IND

**Keywords:** ​​​​​​atherosclerosis, ​coronary artery disease, lp-pla2, pla2g7, r92h, rs1805017, single nucleotide polymorphism

## Abstract

Background

Lipoprotein-associated phospholipase A2 (Lp-PLA2) is a key enzyme selectively expressed in unstable, rupture-prone atherosclerotic plaques. Previous research has established a strong link between the *PLA2G7* gene and the development of coronary artery disease (CAD). While traditional risk factors like cholesterol levels and blood pressure are valuable, there remains a need for more specific biomarkers to identify individuals at heightened risk of atherosclerosis before the onset of clinical symptoms. Our study aimed to investigate the association between serum Lp-PLA2 levels, lipid parameters, cardiac markers, and the rs1805017 variant within the *PLA2G7* gene. By exploring these factors, we sought to enhance the assessment of CAD risk and severity.

Materials and methods

It is a Cross-sectional, case-control study, that recruited 125 subjects, comprising 75 angiographically proven CAD cases and 50 age-sex and ethnically matched controls of a South Indian population. Serum biomarkers were processed according to standard commercial kits. A group of 100 subjects underwent genotyping using Sanger's sequencing method.

Results

Out of the total study population, 25% of the patients were young men (<45 years). We quantitatively estimated the serum Lp-PLA2 levels and evaluated any possible association of serum Lp-PLA2 levels with all the genotypes of R92H (rs1805017) located on Exon 4 of the PLA2G7 gene on chromosome 6. In CAD patients, we found a significant positive correlation of the lipid profile, Lp(a), hs Troponin I, and Lp-PLA2, but a negative correlation with high-density lipoprotein cholesterol (HDL-C) levels. The genotype frequency distributions of the R92H (rs1805017) polymorphisms were GG (17.9%), GA (35.7%), and AA (46.4%) in the control group, and GG (20.3%), GA (30.4%), and AA (49.3%) in CAD cases. The prevalence of the homozygous genotype and serum Lp-PLA2 levels was highest in patients with triple vessel disease (TVD). After correction, logistic regression showed homozygosity as the main independent risk predictor of CAD (p=0.0087). Receiver operating characteristic (ROC) curves revealed that among all biomarkers tested, Lp-PLA2 showed a higher area under the curve (AUC: 0.935), indicating excellent diagnostic ability with a cutoff >392 ng/ml and a sensitivity of 88.2% and specificity of 90.9% in predicting the risk and severity of CAD.

Conclusions

Elevated serum Lp-PLA2 levels and homozygosity of rs1805017 were significantly associated with the risk and severity of disease (TVD). Single nucleotide polymorphism (SNP) analysis can be used as a risk stratification test. Alternatively, as elevated Lp-PLA2 is an indicator of unstable rupture-prone plaques, it can be used as an economical, noninvasive risk and severity predictor, especially in places where coronary angiography (CAG) is not available. Although further research is warranted, these findings can assist in primordial prevention or prescribing a prompt therapeutic algorithm to decrease disease mortality.

## Introduction

Coronary artery disease (CAD) remains a significant global health burden, causing substantial morbidity and mortality [1]. The diagnosis of atherosclerosis often occurs after the onset of symptoms or complications due to coronary artery blockage. While the lipid profile and coronary angiography (CAG) are valuable tools, there is still a need for additional biomarkers to identify individuals at risk of atherosclerosis, especially in asymptomatic populations and in regions with limited access to advanced diagnostic procedures like angiography. Identifying novel predictive blood markers could revolutionize early diagnosis and preventive strategies. By detecting atherosclerosis at an early stage, we can implement timely interventions to mitigate the risk of acute myocardial infarction (AMI) and reduce the need for invasive procedures such as percutaneous transluminal coronary angioplasty (PTCA) and coronary artery bypass graft (CABG), ultimately improving patient outcomes and reducing healthcare costs. We hypothesized that inflammatory markers could enhance cardiovascular risk assessment and enable early intervention, leading to better patient care.

While cardiac troponin is highly sensitive in detecting myocardial necrosis, its specificity is limited as it cannot differentiate between various causes of myocardial injury, including AMI, acute pulmonary embolism, heart failure, myocarditis, or end-stage renal disease [2]. This lack of specificity hinders its ability to definitively identify CAD.

Lipoprotein-associated phospholipase A2 (Lp-PLA2) is a promising biomarker of vascular-specific inflammation that can help clinicians identify rupture-prone atherosclerotic plaque inflammation and assess its stability [3]. This enzyme plays a crucial role in the initiation and progression of atherosclerosis by hydrolyzing oxidized phospholipids on low-density lipoprotein (LDL) within arterial plaques. Lp-PLA2 expression is primarily localized to plaque areas with significant lipid accumulation, leukocyte infiltration, cellular necrosis, and calcification [4]. Importantly, Lp-PLA2 levels are not significantly affected by systemic inflammation, making it a more specific marker of vascular inflammation [5]. Approximately 85% of serum Lp-PLA2 is associated with LDLs. Elevated levels of Lp-PLA2 are indicative of rupture-prone plaques and have been shown to be a strong independent predictor of cardiovascular risk, surpassing the predictive power of C-reactive protein (CRP) alone [6].

Numerous studies have implicated genetic factors in the development of CAD, particularly in Southeast Asian Indian populations. Single nucleotide polymorphisms (SNPs) in the *PLA2G7* gene have emerged as potential genetic risk factors that may increase susceptibility to atherosclerosis and subsequent CAD [7].

This study aims to investigate the association between the *PLA2G7* (R92H) gene polymorphism and serum Lp-PLA2 levels with the risk and severity of CAD.

## Materials and methods

Recruitment of subjects

Study Design

In our case-control cross-sectional study, a total of 125 subjects were recruited, including 75 angiographically proven CAD cases (ages 18-70 years) and 50 age-, sex-, and ethnicity-matched healthy controls, from a tertiary care center at Nizam’s Institute of Medical Sciences (NIMS), Hyderabad, Telangana, India. The cases involved patients with prolonged chest pain for more than 30 minutes, with either ST-elevation myocardial infarction (STEMI) or non-ST-elevation myocardial infarction (NSTEMI), elevated cardiac enzymes, routine blood investigations, 2D echocardiography, and angiographically proven CAD with at least 50% coronary artery stenosis. Cases were divided into groups based on the number of vessels involved, such as single vessel disease (SVD, n=21), double vessel disease (DVD, n=29), and triple vessel disease (TVD, n=25).

Exclusion Criteria

Individuals using lipid-lowering drugs (statins, fibrates, nicotinic acid, or ezetimibe) within the last three months, those with primary myocardiopathy, endocarditis, severe valvular heart disease, any autoimmune disease, another acute or chronic systemic disease, or a malignant tumor were excluded. Healthy controls consisted of 50 individuals matched by age, gender, and ethnicity. Out of the total 125 samples, 100, comprising 70 CAD patients and 30 controls, were further analyzed using DNA sequencing for the *PLA2G7* (rs1805017, R92H) gene, and grouped into wild (GG), heterozygous (GA), and homozygous (AA) variants. Informed consent was obtained from all study subjects. Three milliliters of venous fasting blood samples were collected in plain tubes without any anticoagulant, and serum was aliquoted and stored at -40°C until further analysis. Another 3 ml of venous whole blood sample was collected in an ethylenediaminetetraacetic acid (EDTA) tube for DNA isolation.

Biochemical Analysis

**Figure 1:**
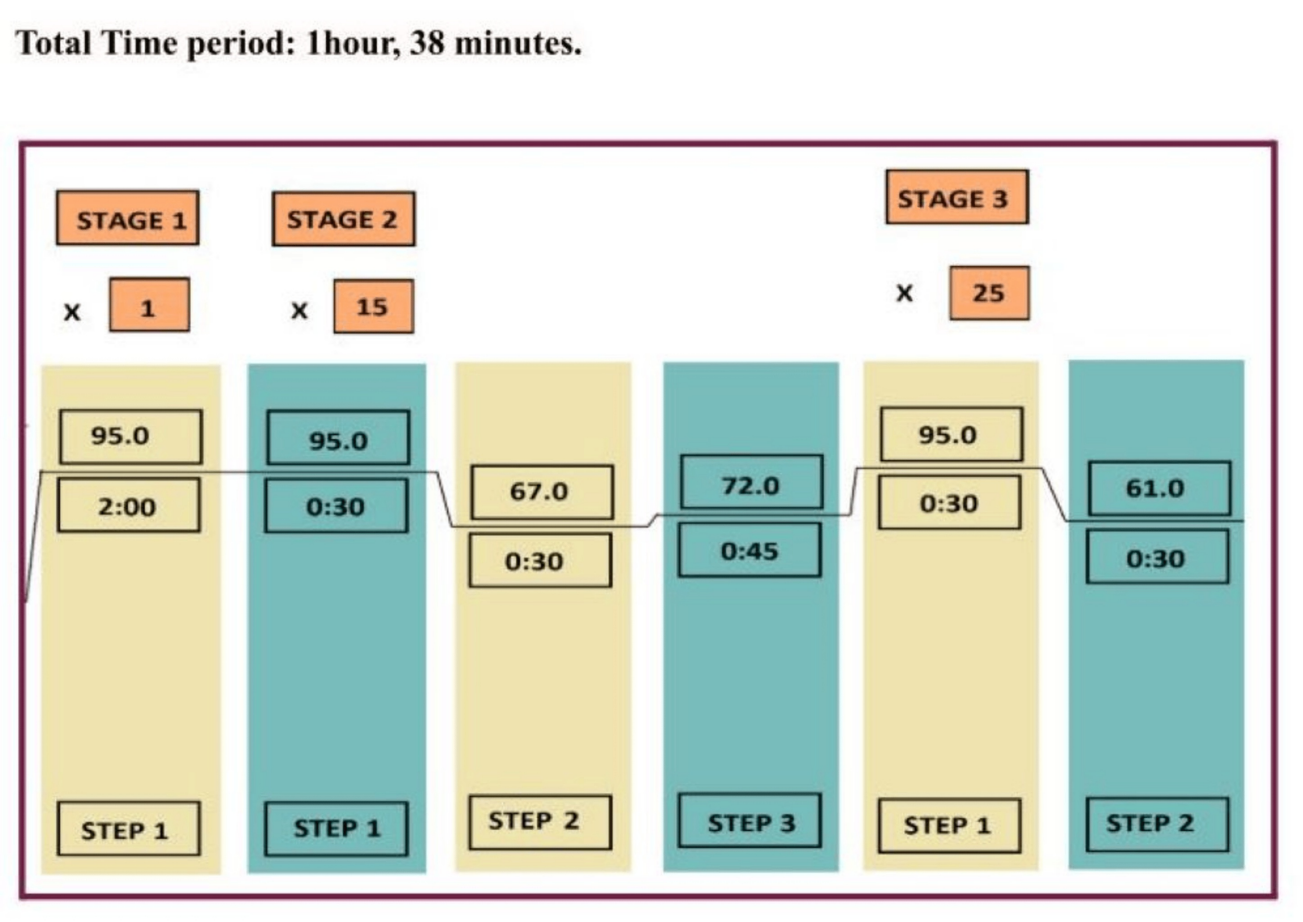
Polymerase chain reaction (PCR) conditions standardized for DNA amplification.

Biochemical analysis of all studied parameters was performed using commercially available kits and a fully automated autoanalyzer (Cobas C501). Serum Lp(a) levels were determined by immunoturbidimetry. The quantitative estimation of serum Lp-PLA2 was performed using a commercially available ELISA Kit (GENLISA ELISA kit from Krishgen, California, USA). This study, carried out following the principles of the Declaration of Helsinki for guiding human research, was approved by the Institutional Ethical Committee of Nizam’s Institute of Medical Sciences, Hyderabad, India (EC/NIMS/2740/2021). *Molecular Workflow* Blood samples were collected in an EDTA vacutainer, and genomic DNA was extracted from peripheral blood leukocytes using a QIAGEN DNA Isolation Kit, as per the manufacturer's protocol. The concentration and purity of DNA samples were determined using a Nanodrop 2000c instrument (ThermoScientific, USA). Absorbance was taken at 260 nm and 280 nm. DNA free from contaminant protein/phenol/RNA showed an A260/A280 ratio of 1.8-2.0 in 10 mM Tris Cl, pH 8.5. An optical density of 1.00 corresponds to approximately 50 ng/μl of double-stranded DNA. The concentration of DNA in ng/μl is calculated as A260 × 50 × dilution factor. Strong absorbance at A280, resulting in a low A260/A280 ratio, indicates the presence of contaminants, such as proteins. Strong absorbance at 270 nm and 275 nm indicated the presence of contaminating phenol. Absorbance at 325 nm suggested contamination by particulates in the solution or a dirty cuvette. Purity was checked for all such samples, and DNA was reisolated if required, with purity rechecked. Spectrophotometric methods may not be useful when DNA concentration/amount is not sufficient. Thus, in all cases, a mini gel was run, and the quality of DNA in samples was determined by comparing the fluorescent yield of the unknown sample with that of standard DNA. Genotyping of the *PLA2G7* gene for (rs1805017), R92H variants, was amplified and sequenced in both directions using the following primers:

Forward: 5'-AGATAATGATCCCTTGAC-3’; Reverse: 5'-GTCAAGGGATCATTATCT-3'. Each PCR reaction contained 50-100 ng genomic DNA, 2.0 pmol of each primer, and 5 μl of Takara EmeraldAmp GT PCR master mix in a final volume of 10 μl, and the reaction was carried out using an Eppendorf Mastercycler. The PCR conditions are as follows (Figure 1): Step 1: Initial denaturation (Stage 1), 1 cycle at 95° C for 2 minutes followed by DNA denaturation (Stage 2), 15 cycles at 95°C for 30 seconds. Step 2: Annealing at 67°C for 30 seconds. Step 3: Elongation at 72°C for 45 seconds. After 15 cycles, another 25 cycles were run under the following PCR conditions: Stage 3: Denaturation at 95°C for 30 seconds, Annealing at 61°C for 30 seconds, Elongation at 72°C for 45 seconds, and Final Extension at 72°C for 5 minutes.

**Figure 2:**
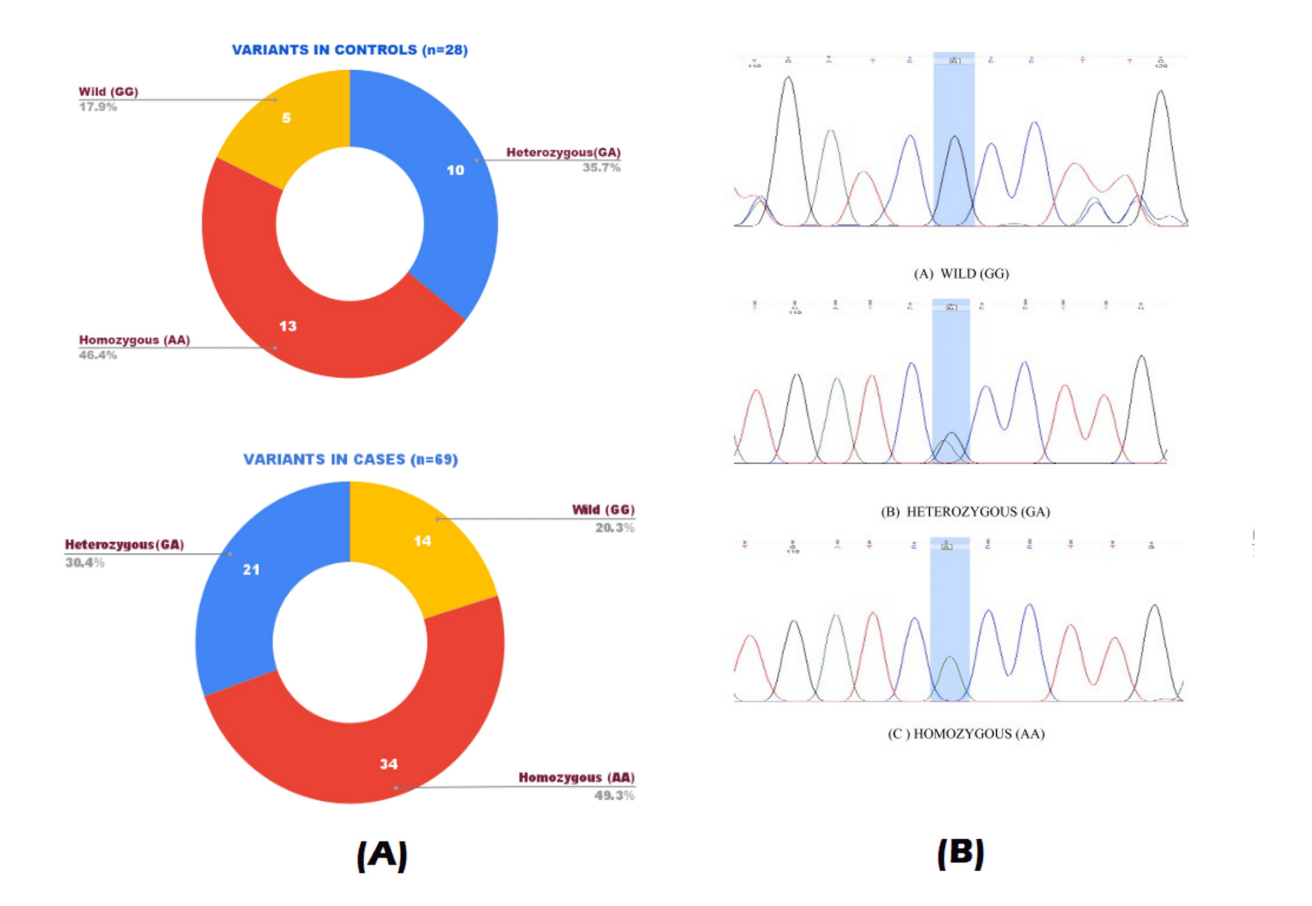
(A) Distribution of single nucleotide variations in the total study population; (B) Automated DNA sequencing chromatograms representing the rs1805017 (G to A transition). (2B) A four-color sequencing chromatogram generated by an automated sequencing machine displays the results of a sequencing run, where each of the nitrogenous bases is indicated by a specific color: adenine (green), cytosine (blue), guanine (black), and thymine (red). (A) Wild variant (G/G), (B) Heterozygous variant (G/A), (C) Homozygous variant (A/A).

The amplified PCR fragments were sequenced using the Big-Dye Terminator v3.1 Cycle Sequencing Kit (Applied Biosystems) on an ABI Prism 3730XL Genetic Analyzer (Applied Biosystems), according to the manufacturer’s instructions. Sequencing chromatograms, as shown in Figure 2, were visualized using FinchTV software, and variations were noted after comparing them with reference sequences (ENSG00000232810).

Statistical analysis

The quantitative data were presented as mean ± SD, and qualitative data (clinical phenotypes) were expressed as percentages. *PLA2G7* gene (rs1805017) R92H variants were tested for deviation from Hardy-Weinberg equilibrium using the chi-square (χ2) test. The genotypes; homozygous for polymorphism (AA), heterozygous for the polymorphism (GA), and wild type (GG), were analyzed in our study population. A predominantly dominant model (GG vs GA+AA) was used to ascertain statistical significance. Normally distributed variables were analyzed by ANOVA test. Non-normally distributed variables were analyzed by Kruskal-Wallis test. A p-value of <0.05 was taken as statistically significant. A simple gene counting method determined genotype and allele frequencies between cases and controls. Fisher’s exact two-tailed test was performed to determine statistical significance, and P values less than 0.05 were considered significant. The odds ratios (OR) were calculated from genotype and allele frequencies at a 95% CI using the online tool Vassar Stats Calculator (www.faculty.vassar.edu/lowry/VassarStats.html). The statistical analysis was done using Microsoft Excel, GraphPad Prism version 9.4.1, and MedCalc Statistical Software version 20.115.

## Results

The mean age of controls and cases are 50.62 ± 8.05 and 53.44 ± 9.1 years, respectively. There was no statistical difference between controls and cases in terms of age (p=0.092) and gender distribution (p=0.511). Family history was found to be significantly associated with heart disease (p=0.02). In CAD cases, the incidence of hypertension, diabetes, smoking, and alcohol intake were found to be high. However, there was no significant difference between the two groups. Serum total cholesterol, triglycerides, and LDL-C were significantly higher (p<0.05), and serum HDL-C was significantly lower (p<0.05) in cases compared to controls. We further studied the levels of serum Lp(a) and Lp-PLA2 in controls and cases and observed a significant increase in the levels of serum Lp(a), Troponin I, and Lp-PLA2 in cases compared to controls (Table 1).

**Table 1:** Multivariate analysis of study participants. *Mean ± SD for normal distribution, ^Median and IQR for non normal distribution. The unpaired t-test was used for comparison of normal data; the Mann-Whitney U-test for non-normal data, and Fisher’s exact test for comparison of proportions. P<0.05 was considered statistically significant. HDL: High-density lipoproteins; LDL: Low-density lipoprotein; Trop-I: Troponin I; LP-PLA2: Lipoprotein-associated phospholipase A2; IQR: Interquartile range.

Parameter	Controls (n=50), n (%)	Cases (n=75), n (%)	P-value
Age (years)*	50.62 ± 8.05	53.44 ± 9.1	0.092
Male: Female n(%)	70:30	76:24	0.511
Smoking n(%) Yes/No	10 (20)/40 (80)	25 (33)/50 (67)	0.819
Tobacco n(%) Yes/No	15 (30)/35 (70)	32 (43)/43 (57)	0.867
Alcohol intake n(%) Yes/No	16 (32)/34 (68)	41 (55)/34 (45)	0.88
Hypertension n(%) Yes/No	18 (36)/32 (64)	33 (44)/42 (56)	0.746
Diabetes mellitus n(%) Yes/No	10 (20)/40 (80)	24 (32)/51 (68)	0.203
Family History n (%) Yes/No	15 (30)/35 (70)	45 (60)/30 (40)	0.002*
Serum Total Cholesterol ^ (mg/dl)	152.5 (143-173.5)	189 (165.5 -220)	<0.0001*
Serum HDL-C^ (mg/dl)	35 (28-39)	22 ( 19-27 )	<0.0001*
Serum LDL-C^ (mg/dl)	117.5 (104.5-123)	154 (130-167)	<0.0001*
Serum Triglycerides^ (mg/dl)	115.5 (105-133.5)	157 (135-185)	<0.0001*
Serum Lipoprotein (a)^ (mg/dl)	18 (14-23.5)	52 (37-64 )	<0.0001*
Trop- I ^ (ng/L)	14.5 (8.25-20)	93 (71.5-130)	<0.0001*
LP-PLA2* (ng/ml)	288.02 ± 80.6	458.2 ± 131.97	<0.0001*

Further, the data were stratified into disease phenotypes such as single, double, and TVD, and the results are depicted in Table 2. We found a significant increase in serum total cholesterol, HDL-C, LDL-C, triglycerides, CK-MB, Troponin, LDH, LP(a), and Lp-PLA2 levels in patients with TVD compared to single (SVD) and double vessel disease (DVD). These levels increased from single to double to TVD (Table 2).

**Table 2:** Biochemical data based on disease phenotype. *Mean ± SD is used because the data are normally distributed. One-way ANOVA is used for comparison between groups. Median (IQR)# is used for data that are not normally distributed. The Kruskal-Wallis test is used for comparison between groups. *p < 0.05 is considered significant. SVD: Single Vessel Disease; DVD: Double Vessel Disease; TVD: Triple Vessel Disease; HDL: High-Density Lipoproteins; LDL: Low-Density Lipoprotein; CK-MB mass: Creatine Kinase-MB mass; LDH: Lactate Dehydrogenase; Trop-I: Troponin I; Lp(a): Lipoprotein (a); LP-PLA2: Lipoprotein-Associated Phospholipase A2; IQR: Interquartile Range.

Parameters	Controls (n=50) (Median (IQR)	SVD (n=21) (Median (IQR)	DVD (n=29) (Median (IQR)	TVD (n=25) (Median (IQR)	P-value
Total Cholesterol^ (mg/dl)	152.5 (143-173.5)	210 (171-243.5)	181 (156.5- 207)	189 (167-213)	0.0009*
HDL-C (mg/dl) ^	35 (28-39)	21.3 (17.5- 25)	21.2 (20-27)	22.8 (17-28)	<0.0001*
LDL (mg/dl) *	69 ± 12.5	147 ± 21.4	150 ± 21.87	150 ± 21.83	<0.0001*
Triglycerides (mg/dl) ^	115.5 (105-133.5)	176 (124.5-199)	157 (136.5-185)	151 (130.5-173.5 )	<0.0001*
CK-MB Mass (ng/ml) ^	4.0 (2.1 -5.0)	9.8 (6.35- 12.9)	8.1 (6.4-11)	11.2 (6.95-13.5)	<0.0001*
LDH (IU/L)^	179.5 (145.25-203.75)	277 (191.5-475.5)	355 (244-437)	338 (265-365.5)	<0.0001*
Trop-I (ng/L)^	14.5 (8.25-20)	89 (65.5-167.5)	95 (71.5-136.5)	97 (73.5-123)	<0 .0001*
Lp(a) (mg/dl) ^	18 (14-23.5)	52 (37.5- 64)	55 (32.5-65.5)	42 (37-60)	<0.0001*
LP-PLA2* (ng/ml)	288.02 ± 80.65	322 ± 70.28	442.04 ± 43.03	599.1 ± 87.5	<0.0001*

Molecular workflow

Out of the total sample size (n=125), DNA from 100 whole blood samples was sequenced for the *PLA2G7* gene polymorphism (R92H) (rs1805017) using Sanger’s dye termination DNA sequencing method. Of these, 70 were cases and 30 were controls. Due to unknown reasons, we couldn't obtain DNA sequences from 1 case and 2 controls (Sample size = 97). The genotype frequency distributions of the R92H (rs1805017) polymorphisms were GG (17.9%), GA (35.7%), and AA (46.4%) in the control group and GG (20.3%), GA (30.4%), and AA (49.3%) in CAD cases, respectively (Figure 2).

Based on the simple allelic frequency distribution counting method, the (A) allelic frequency of total study participants = 125 (64.5%) was higher than the (G) allelic frequency of total study participants = 69 (35.5%). A Chi-square test of independence showed that there was a random selection and allelic distribution following Hardy-Weinberg equilibrium (p =0.5) in controls. In cases, there was a non-random selection of CAD cases (p=0.02). The data from the “Aggregate allele and genotype frequencies computed from non-sensitive dbGaP studies (ALFA)” project was used to deduce minor allele frequencies. Data was obtained from the 1000 Genome Project. A Chi-square test was carried out using our data as a baseline to compare the distribution with other populations' variant allele, Homozygous allele, OR odds ratio, CI confidence interval * p < 0.0001 (OR-35.83, 95% CI (22.72-56.5)) with the Asian population and 0.03 (OR-2.38, 95% CI (1.36-4.18)) with Indian Telugu living in the USA.

Prevalence of *PLA2G7* variants in CAD subgroups 

A Chi-square test of independence was performed to examine the relationship between the distribution of Wild, Heterozygous, and Homozygous groups of Cases. The distribution between the groups was highly significant (p = 0.0006). Our study also observed that, among the Homozygous cases, TVD cases were more than SVD cases. Among the Wild cases, SVD cases were more than TVD cases. This indicates that the *PLA2G7* Homozygous variants in CAD patients are associated with the involvement of more coronary artery vessels.

Genetic influences on serum Lp-PLA2 levels

There was a significant increase in serum Lp-PLA2 levels between the variants of controls and cases (p = 0.0023). Among the variants, the homozygous (AA) group in cases had significantly elevated levels of Lp-PLA2 compared to controls, wild (GG), and heterozygous (GA) variants, as shown in Table 3.

**Table 3:** Mean concentration of serum Lp-PLA2 (ng/ml) in R92H variants in controls and cases. Mean ± SD is calculated for normally distributed data. The independent Student's t-test was used. p < 0.05 is considered significant.

Variants	LP-PLA2 (ng/ml) Control	LP-PLA2 (ng/ml) Cases	P-value
Wild	249.2 ± 12.72	351.8 ± 135.8	0.05 *
Heterozygous	329.56 ± 81.53	385.4 ± 120.5	0.027 *
Homozygous	323 ± 122.4	597.4 ± 125.8	0.0001 *

**Figure 3:**
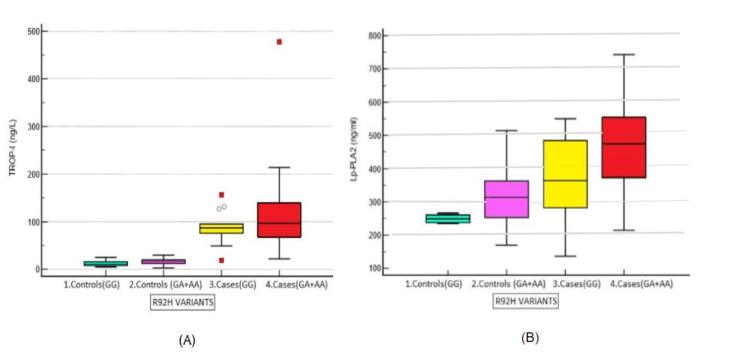
(A) Comparison of Hs-Trop I (ng/L) levels among R92H variants; (B) Comparison of Lp-PLA2 (ng/ml) levels among R92H variants in the dominant model. 3(A) In the post-hoc analysis, there was a significant difference in the levels of serum Hs-Trop I between controls and case groups (p < 0.0001). However, there was no statistically significant difference in the intergroup comparison (p > 0.999). 3(B) Serum Lp-PLA2 levels were significantly elevated in cases (p < 0.0001) and also showed a significant elevation between subgroups (p < 0.0001) when compared to controls. This indicates that serum Lp-PLA2 levels increase with the involvement of more coronary vessels, making it a better severity assessment marker than Hs-Trop I.

Lp-PLA2 post-hoc analysis (Tukey’s) revealed significant differences when multiple comparisons were made between controls and cases. However, no significant difference was found between Wild cases and controls (p > 0.05). For other parameters, there were significant differences between controls and cases (p < 0.05) but not between subgroups. Figure 3 compares Lp-PLA2 and Hs-Trop I levels.

**Figure 4:**
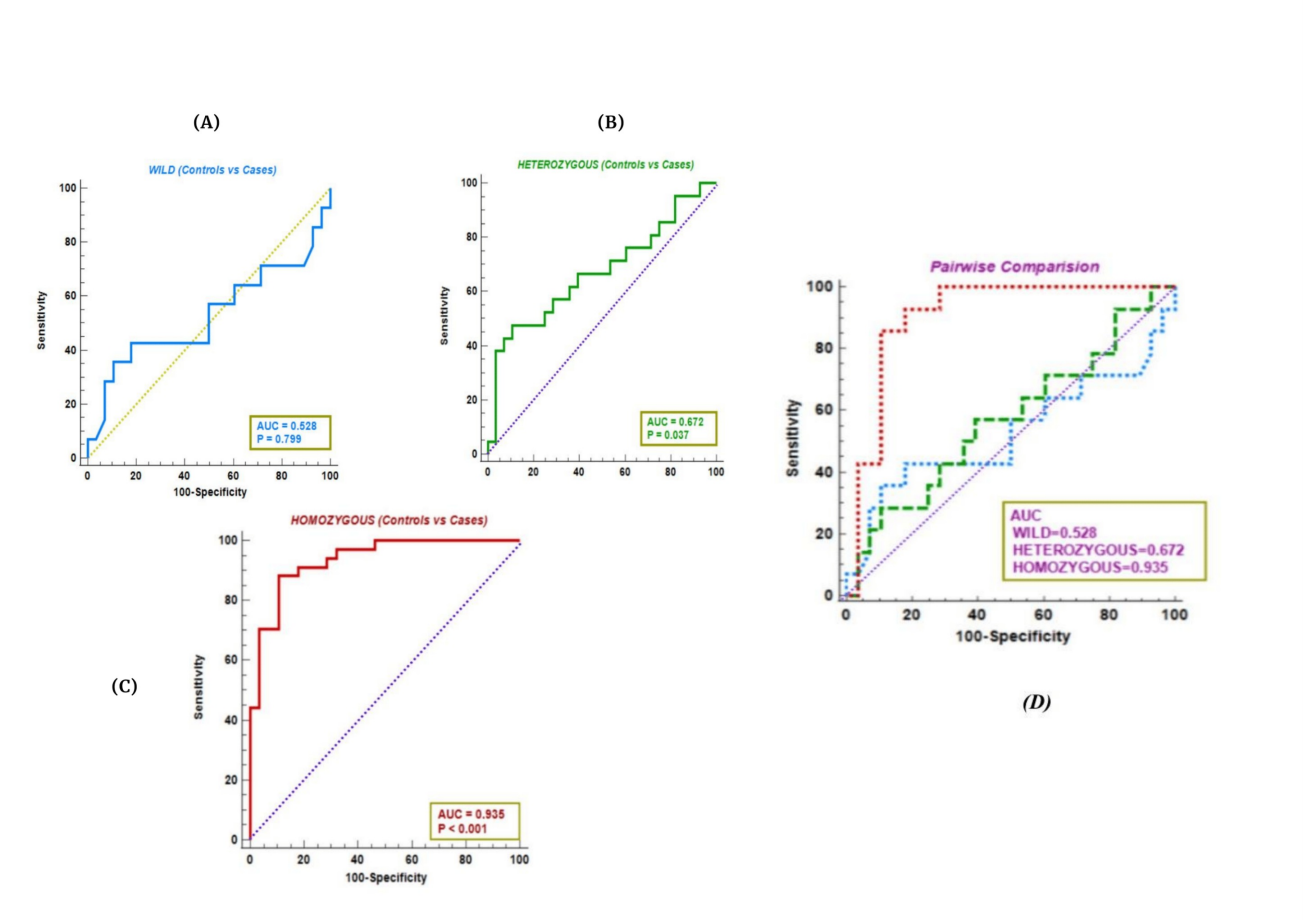
Diagnostic ability and severity assessment of serum Lp-PLA2 in R92H variants. A, B, C, and D represent ROC curves (sensitivity vs. 1-specificity) for high-risk CAD in wild, heterozygous, homozygous, and pairwise comparisons, respectively. The area under the curve (AUC) was used to discriminate between cases and controls.

To assess the diagnostic ability and severity assessment of serum Lp-PLA2 in *PLA2G7* (R92H) variants, receiver operating characteristic (ROC) analysis was conducted. The area under the curve was used to discriminate between cases and controls, as shown in Figure 4. In the total study population, controls vs. cases, in wild controls vs. cases (A), an AUC of 0.528 at a cutoff >375 ng/ml with 42.9% sensitivity and 82.1% specificity showed poor predictability. In heterozygous controls vs. cases (B), AUC = 0.672, at a cutoff of > 392 ng/ml, serum Lp-PLA2 showed a sensitivity of 47.6% and a specificity of 89.3%, indicating moderate predictability. In homozygous controls vs. cases (C) at AUC = 0.935 and a cutoff of > 392 ng/ml, Lp-PLA2 demonstrated a sensitivity of 88.2% and a specificity of 90.9%, showing excellent predictability of the disease as presented in Figure 4.

When multiple logistic regression analysis of the study population was performed without considering genetic polymorphism as a risk factor, Lp-PLA2 was found to be an independent risk predictor of CAD along with gender, smoking, diabetes, and hypertension. The odds ratio (OR) for Lp-PLA2 is 1.0238 (cases versus control group, 95% CI 1.0136 -1.0341, P < 0.0001). However, after correction for age, gender, and other risk factors, when genetic variants were included as one of the risk factors of CAD, the homozygous variant (AA) was found to be the only independent risk predictor for the development of CAD (odds ratio of 1.0094, 95% CI of 1.0023 to 1.0165; p= 0.0092) in stepwise backward logistic regression (Table 4). Using Spearman's correlation, all lipid parameters and cardiac markers were found to positively correlate with Lp-PLA2 except for HDL-C, which was negatively correlating. In our study, we correlated serum Lp-PLA2 levels with total cholesterol, TG, HDL-C, LDL, and other baseline characteristics. Total cholesterol (Spearman’s r = 0.319, p= 0.0001), TG (Spearman’s r = 0.324, p= 0.0001), LDL cholesterol (Spearman’s r = 0.396, p= 0.0001), and Lp(a) (Spearman’s r = 0.504, p= 0.0001). There was a negative correlation of HDL-C (Spearman’s r = -0.409, p= 0.0001) with serum Lp-PLA2 levels.

**Table 4:** Stepwise multiple logistic regression analysis for risk stratification of CAD (p<0.05 is significant). CAD: Coronary artery disease.

Variable	Coefficient	Standard Error	Wald	P-value
Homozygous	0.0092	0.0035	6.88	0.0087*
Lp-PLA2	0.0038	0.0047	0.67	0.41
Age	0.048	0.0568	0.72	0.39
Smoking	0.16	1.25	0.018	0.89
Alcohol	0.56	1.087	0.23	0.62
Family History	0.13	1.026	0.017	0.89
Diabetes	0.11	1.175	0.0095	0.92
Constant	29.25	7283.12	0.000016	0.99

## Discussion

Previous studies have yielded conflicting results regarding the association between *PLA2G7* polymorphisms and the risk of CAD. While some studies have demonstrated an association between *PLA2G7* polymorphism and CAD risk [8], others have suggested an antiatherogenic or protective role of the *PLA2G7* gene encoding Lp-PLA2 [9]. To our knowledge, no prior research has directly examined the relationship between serum Lp-PLA2 levels, cardiac markers, and *PLA2G7* genetic variants in the context of CAD.

Given these knowledge gaps, our pilot study aimed to investigate the impact of the *PLA2G7* R92H polymorphism on clinical atherosclerosis. This missense variant, located on chromosome 6, exon 4, has been of particular interest. Our study is the first to explore the association between *PLA2G7* genetic variants, serum Lp-PLA2 concentrations, cardiac markers, and lipid profiles in CAD. By comparing and correlating serum Lp-PLA2 levels with established risk factors, comorbidities, and biomarkers associated with CAD, we sought to contribute to the development of primordial and primary preventive strategies for this disease.

The demographic data and baseline characteristics of the study participants did not reveal significant differences between the cases and controls, indicating that both groups were drawn from a similar population. A notable finding was the presence of 19 cases (25%) below the age of 45, including a 21-year-old male with a history of alcohol addiction and a family history of CAD. While various studies have defined young CAD within age ranges of 35 to 55 years [10], our study highlights the potential for earlier onset, particularly in individuals with risk factors.

Our analysis revealed a positive correlation between serum Lp-PLA2 levels and traditional cardiovascular risk factors: total cholesterol (Spearman's r = 0.319, p = 0.0001), triglycerides (TG) (Spearman's r = 0.324, p = 0.0001), LDL cholesterol (Spearman's r = 0.396, p = 0.0001), and Lp(a) (Spearman's r = 0.504, p = 0.0001). Conversely, a negative correlation was observed with HDL-C (Spearman's r = -0.409, p = 0.0001). These findings align with well-established knowledge. Low HDL-C levels are consistently associated with an increased risk of CAD, as demonstrated in numerous case-control and prospective observational studies, including the Framingham Heart Study, which observed that the risk for CAD increased sharply as HDL levels fell progressively below 40 mg/dL [11]. In contrast, higher HDL-C levels are linked to reduced cardiovascular risk and increased longevity.

In the present study, a significant association was found between elevated Lp-PLA2 levels and the *PLA2G7* R92H polymorphism in CAD patients. The homozygous variant (AA) was the most prevalent genotype (48.45%), surpassing the wild-type genotype (GG, 19.58%). The altered A allele (64.43%) demonstrated a higher frequency compared to the reference G allele (35.57%).

Our findings align with previous studies in the SNPedia database [12], such as those conducted in Gujarati Indians in Houston, Texas, and Chinese populations. While the Chinese study on 544 subjects reported a higher prevalence of the wild-type genotype [13], other studies, including those involving the CATHGEN and GENECARD families, have shown an association between the homozygous AA genotype and an increased risk of CAD. This association is likely attributed to higher Lp-PLA2 concentrations (p = 0.0023) in individuals with the AA genotype [14].

To evaluate the diagnostic accuracy of Lp-PLA2 in individuals with the R92H genetic polymorphism, we conducted a ROC analysis. Similar to the findings reported by Ma S et al. [13], who observed an AUC of 0.825 (95% CI: 0.722-0.929) for Lp-PLA2 in the acute coronary syndrome (ACS) group, we found that Lp-PLA2 exhibited an AUC of 0.935 (95% CI: 0.84-0.98), with a sensitivity of 88.2% and specificity of 90.9% at a cutoff value of >392 ng/ml in homozygous variant carriers. These results suggest that the R92H polymorphism may be a significant risk factor for CAD.

After adjusting for age, gender, baseline characteristics, and lipid profile, logistic regression analysis revealed that the R92H homozygous variant (AA) was an independent risk predictor for CAD (odds ratio: 1.0094, 95% CI: 1.0023-1.0165, p = 0.0092). These findings align with previous studies, such as those by Sutton BS et al. [14] and Robert R et al. [15]. Sutton's comprehensive genetic analysis identified R92H and A379V as two of the most significant SNPs associated with CAD, even after adjusting for multiple comparisons [14]. Robert's research highlighted the role of the 9p21 locus, including the R92H variant, in elevating CAD risk. Individuals with the homozygous genotype at this locus have a significantly higher risk compared to those with the wild-type genotype. In the overall population, the heterozygote is associated with a 25% increased risk, and the homozygous genotype with a 50% increased risk [15].

Our study compared the genetic data of our cases with reference data from the 1000 Genomes Project [16]. Using a chi-square test, we assessed significant differences in the distribution of variant alleles, homozygous alleles, and odds ratios (ORs) with 95% CIs in our population compared to other populations, particularly Asian populations and Indian Telugus residing in the USA. Our findings were consistent with those of Naushad SM et al. [17], who compared their population's NUDT15 and TPMT3C frequencies with European and African/African American populations. Their study also revealed significant differences in allele frequencies across populations.

Our findings indicate that individuals with the homozygous (AA) genotype for the R92H polymorphism have a significantly higher risk of developing CAD compared to those with the wild-type (GG) or heterozygous (GA) genotypes. Moreover, serum Lp-PLA2 levels were significantly elevated in individuals with the AA genotype compared to those with the GG or GA genotypes in CAD patients. These results underscore the association between the R92H polymorphism, elevated Lp-PLA2 levels, and the severity of CAD in angiographically confirmed cases. After adjusting for confounding factors such as age, gender, lipid profile, and other risk factors, the homozygous genotype remained an independent predictor of CAD.

Limitations

However, due to budget constraints, polymorphism analysis for cluster genes was not feasible. The cross-sectional study design and single-center data collection limited the generalizability of the findings. The lack of randomization and follow-up further restricts the ability to draw definitive conclusions about the population at large. Further research with larger cohorts is warranted to confirm these findings and explore the underlying mechanisms.

## Conclusions

Our pilot study is a pioneering effort to investigate the association between the *PLA2G7* gene polymorphism (R92H) and various cardiovascular risk factors, including serum Lp-PLA2, cardiac markers, and lipid profiles, in relation to the extent of CAD. Our findings highlight the implications of early intervention, as the elevated homozygous frequency of the *PLA2G7* (rs1805017) variant and increased serum Lp-PLA2 activity observed in CAD patients, particularly those with TVD, underscore the role of Lp-PLA2 in assessing CAD risk and severity. While CAG remains the gold standard for diagnosing CAD, it is an invasive procedure. In contrast, measuring serum Lp-PLA2 offers a non-invasive approach that could aid in the diagnosis, risk stratification, and severity assessment of CAD.

Future research should delve deeper into the complex interplay between genetic and environmental factors by exploring gene-gene and gene-environment interactions involving the *PLA2G7* gene and other relevant genes. Additionally, large-scale studies are needed to identify novel polymorphisms and mutations associated with CAD. By advancing our understanding of the genetic and biochemical mechanisms underlying CAD, we may develop more effective strategies for prevention, early detection, and targeted treatment of this debilitating disease.
